# Non-rigid registration of medical images based on ${S}_2^1\left({\Delta}_{mn}^{(2)}\right)$ non-tensor product B-spline

**DOI:** 10.1186/s42492-022-00101-8

**Published:** 2022-02-02

**Authors:** Qi Zheng, Chaoyue Liu, Jincai Chang

**Affiliations:** 1 College of Sciences, North China University of Science and Technology, Tangshan, 063210 China; 2 Hebei Key Laboratory of Data Science and Application, North China University of Science and Technology, Tangshan, 063210 China

**Keywords:** Non-rigid registration, Diagonal direction, Non-tensor product type B-spline, Boundary triangle domain

## Abstract

In this study, a non-tensor product B-spline algorithm is applied to the search space of the registration process, and a new method of image non-rigid registration is proposed. The tensor product B-spline is a function defined in the two directions of *x* and *y*, while the non-tensor product B-spline ${S}_2^1\left({\Delta}_{mn}^{(2)}\right)$ is defined in four directions on the 2-type triangulation. For certain problems, using non-tensor product B-splines to describe the non-rigid deformation of an image can more accurately extract the four-directional information of the image, thereby describing the global or local non-rigid deformation of the image in more directions. Indeed, it provides a method to solve the problem of image deformation in multiple directions. In addition, the region of interest of medical images is irregular, and usually no value exists on the boundary triangle. The value of the basis function of the non-tensor product B-spline on the boundary triangle is only 0. The algorithm process is optimized. The algorithm performs completely automatic non-rigid registration of computed tomography and magnetic resonance imaging images of patients. In particular, this study compares the performance of the proposed algorithm with the tensor product B-spline registration algorithm. The results elucidate that the proposed algorithm clearly improves the accuracy.

## Introduction

**Fig. 1:**
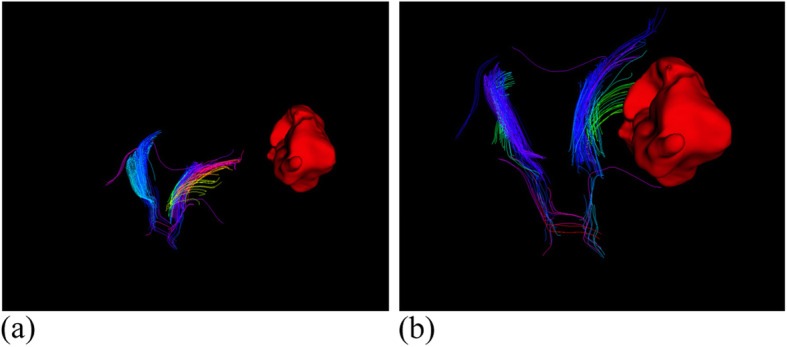
3D model of before and after registration. (**a**): 3D model before registration; (**b**): 3D model after registration

Medical imaging technologies are essential in modern medical diagnosis, and provides indispensable help for doctors to accurately determine the condition of patients’ lesions. At present, the most commonly used medical imaging techniques include computed tomography (CT), magnetic resonance imaging (MRI), positron emission computed tomography (PET), and ultrasound (US). However, in the treatment cycle of each patient, the human tissue inevitably undergoes a local deformation, and doctors cannot accurately judge the changes in the lesions using medical images. The medical image non-rigid registration algorithm can solve this problem to a certain extent, which is a search for the transformation relationship between images collected at different times or using different instruments [1]. According to the existing three-dimensional (3D) modeling technologies [2, 3], Fig. 1 illustrates a 3D model of hematoma and nerve bundle before and after registration, respectively.

In the 1980s, the medical image scanning technology was relatively backward, and most registrations were only applied to rigid registrations in the same modalities. In 1992, Brown [4] classified registration methods according to the complexity of the transformation model and proposed four components of registration, including the feature space, similarity measure, search space, and optimization strategy. In the mid to the late 1990s, with the application of CT, MRI, PET, US, and other medical imaging equipment in clinical diagnosis, doctors and scientific researchers realized that medical images of different modalities can provide various information and a solid basis for diagnosis. Maes et al. [5] applied the concept of mutual information to multimodal medical image registration and used the mutual information between the reference and moving images as a similarity measure for image registration. In 1999, Rueckert et al. [6] proposed a free deformation model based on a B-spline. Subsequently, they applied the uniform multi-level B-spline method of discrete data interpolation, proposed by Lee et al. [7], to the non-rigid registration of breast MRI, which could restore the motion and deformation of the breast [8]. Subsequent methods attempted to improve this method. In the twenty-first century, the rapid development of computer hardware has promoted studies on the image registration in 3D and even four-dimensional fields, particularly on the non-rigid registration between different images of a certain patient. In 2001, Schnabel et al. [9] proposed a non-uniform multilayer B-spline to improve the registration efficiency. In 2013, Oliveira and Tavares [10] introduced a new enhanced B-spline method to register the plantar pressure image sequences in time and space simultaneously. Although the traditional B-spline registration algorithm can achieve reasonable results, it is difficult to obtain accurate results for images with large local and global distortions. The accuracy of the algorithm is low when the smoothing term in the cost function is large. When the smoothing term is considerably small or close to 0, over-registration occurs, severely destroying the image topology. Therefore, some areas cannot be registered. In view of this problem, Ji et al. [11] presented a non-rigid registration algorithm based on a multilevel B-spline, and checked the influence of the balance item using the L2 regularization term, which improved the registration accuracy.

In 1975, Wang [12] established the basic theoretical framework of multivariate splines on arbitrary subdivisions, proposed the smoothing cofactor conformality method, and pioneered the algebraic geometry method for studying multivariate splines. Subsequently, many types of non-tensor product expressions for binary B-spline spaces and basic functions were introduced. In particular, the binary B-spline was on the 1-type and 2-type triangulations. In 1984, Chui and Wang [13] provided a bivariate quadratic first-order smoothness B-spline basis with a minimum symmetric support under the uniform division and constructed an ${S}_2^1\left({\Delta}_{mn}^{(2)}\right)$ non-tensor product B-spline theoretical framework. In 2001, the theory of multivariate spline was organized, while the theory of smooth cofactor coordination method and its application in multivariate spline function were introduced in detail [14]. In addition, some considerable results were achieved in various applications [15–17]. To the best of our knowledge, the application of the above-mentioned splines and registration of medical images has not been reported yet.

The tensor product B-spline in the rectangular domain can only express the information in the horizontal and vertical directions. The direction information is crucially important in the registration of images. The non-tensor product B-spline may contain more directional information, which can display more feature information. In this study, a new registration algorithm is proposed that uses non-tensor product B-spline ${S}_2^1\left({\Delta}_{mn}^{(2)}\right)$ on the 2-type triangulation in search space to describe the global or local motion of the image in more directions [18]. The brain CT and MRI were selected for the registration test. In addition, there are basis functions that can describe the deformation of an image in four directions, which compensates for the lack of tensor product B-spline functions to a certain extent and achieves accurate results.

The remainder of this paper is organized as follows. First, the basic framework of image registration is introduced, and explicit expressions and images of non-tensor product B-spline basis functions in ${S}_2^1\left({\Delta}_{mn}^{(2)}\right)$ space are provided, followed by the registration experiments and a detailed analysis.

## Methods

### Non-tensor product B-spline in the ${S}_2^1\left({\Delta}_{mn}^{(2)}\right)$ space

#### Uniform 2-type triangulation

Uniform 2-type triangulation is a triangulation formed by connecting two diagonal lines of each small rectangle on the basis of a rectangular division. If the rectangular division is uniformly divided, the formation of a uniform 2-type triangulation is also achieved using the division.

**Fig. 2:**
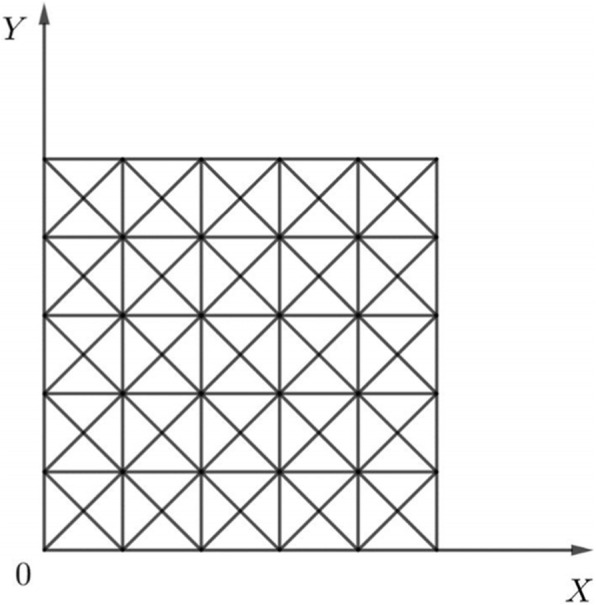
Area *D* and its division ${\Delta}_{mn}^{(2)}$

The uniform 2-type triangulation ${\Delta}_{mn}^{(2)}$ on area *D* is generated by the following division lines, as shown in Fig. 2.

1 $$ {\displaystyle \begin{array}{l} mx-i=0\\ {} ny-i=0\\ {} ny- mx-i=0\\ {} my- nx-i=0\\ {}i=\dots, -1,0,1,\dots \end{array}} $$

#### Basis function of the spline space ${S}_2^1\left({\Delta}_{mn}^{(2)}\right)$ [14]

The dimension of the spline space ${S}_2^1\left({\Delta}_{mn}^{(2)}\right)$ can be expressed as

$$ \dim {S}_2^1\left({\Delta}_{mn}^{(2)}\right)=\left(m+2\right)\left(n+2\right)-1 $$ (2)

**Fig. 3:**
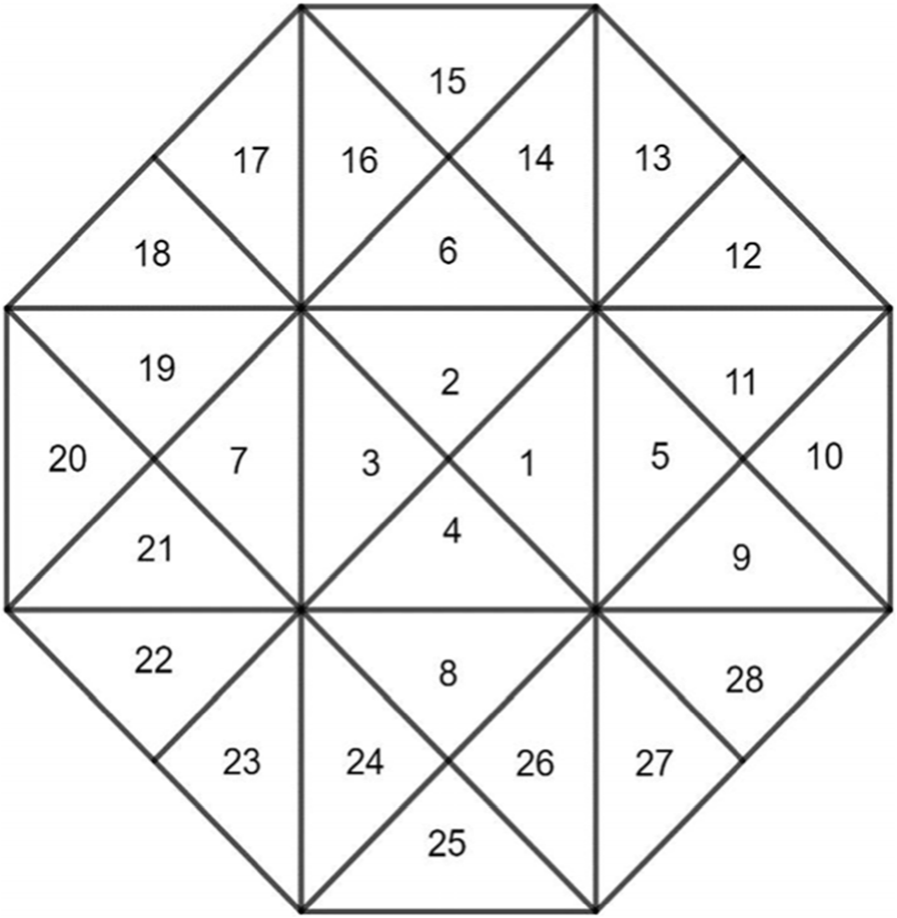
Area *Q* and its division

A local support spline function *B*(*x*, *y*) exists in the space ${S}_2^1\left({\Delta}_{mn}^{(2)}\right)$. Its support is the area *Q* in Fig. 3, where the support function is centered at the origin.

**Fig. 4:**
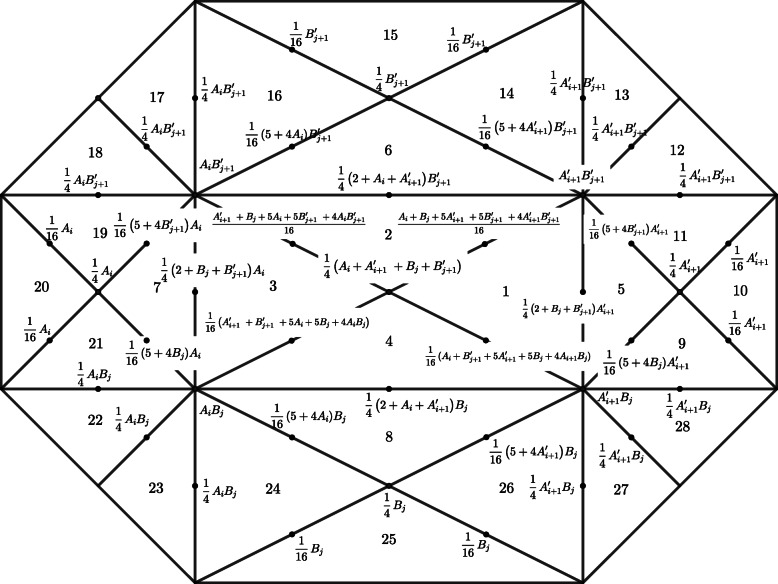
Support of B-spline function *B*(*x*, *y*) on uniform 2-type triangulation

According to the theory of polynomial interpolation, the second-degree polynomial on a triangle can be uniquely determined using the value of the polynomial on the three vertices of the triangle and midpoints of the three sides. In fact, these six points are simply a set of well-posed node groups for the second-degree polynomial interpolation, as shown in Fig. 4.

3 $$ {\displaystyle \begin{array}{l}{A}_i=\frac{x_i-{x}_{i-1}}{x_{i+1}-{x}_{i-1}}\\ {}\;{A}_{i+1}^{\prime }=\frac{x_{i+2}-{x}_{i+1}}{x_{i+2}-{x}_i}\\ {}{B}_j=\frac{y_j-{y}_{j-1}}{y_{j+1}-{y}_{j-1}}\\ {}{B}_{j+1}^{\prime }=\frac{y_{j+2}-{y}_{j+1}}{y_{j+2}-{y}_j}\end{array}} $$

Therefore, it is only necessary to indicate the values of the corresponding six points on the cavities to represent the spline *B*(*x*, *y*) of the above-mentioned local support.

As shown in Fig. 3, for each *i* = 1, …, 28, consider the polynomials as

4 $$ {\displaystyle \begin{array}{l}\begin{array}{l}{p}_1\left(x,y\right)=\frac{1}{2}-\frac{1}{2}{x}^2-\frac{1}{2}{y}^2\\ {}{p}_5\left(x,y\right)=\frac{5}{8}-\frac{1}{2}x-\frac{1}{2}{y}^2\end{array}\\ {}{p}_9\left(x,y\right)=\left(\frac{7}{8}-x+\frac{1}{4}{x}^2\right)+\left(\frac{1}{2}-\frac{1}{2}x\right)y-\frac{1}{4}{y}^2\\ {}{p}_{10}\left(x,y\right)=\frac{9}{8}-\frac{3}{2}x+\frac{1}{2}{x}^2\\ {}{p}_{12}\left(x,y\right)=\left(1-x+\frac{x^2}{4}\right)+\left(-1+\frac{x}{2}\right)y+\frac{1}{4}{y}^2\end{array}} $$

where other polynomials can be obtained using the principle of symmetry.

According to Eq. (4), all polynomials on the cavity are obtained as follows:

5 $$ B\kern0em \left(x,y\right)=\left\{\begin{array}{l}0\\ {}{p}_i\left(x,y\right),i=1,2,\dots, 28\end{array}\right. $$

**Fig. 5:**
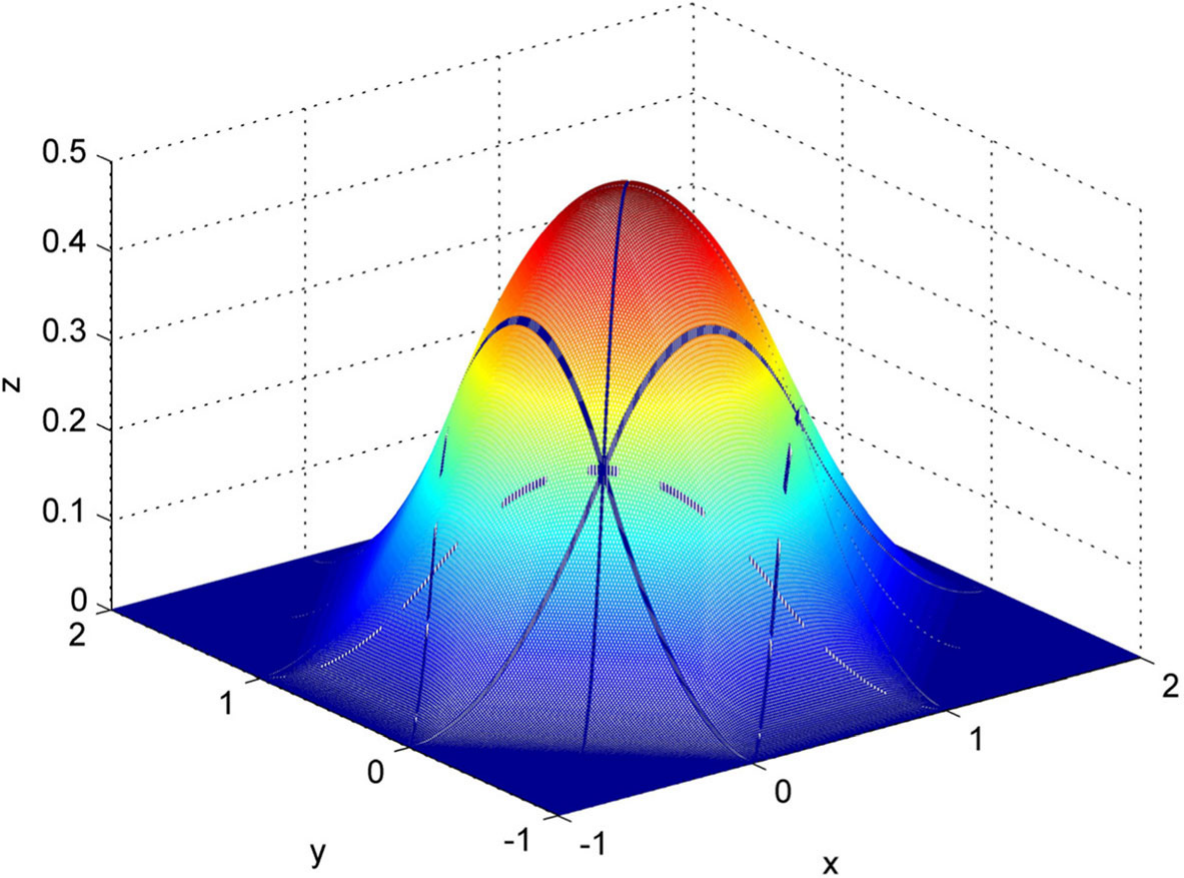
Basis function image

Here, the B-spline basis is ${B}_{ij}\left(x,y\right)=B\left( mx-i+\frac{1}{2}, ny-j+\frac{1}{2}\right)$. Figure 5 demonstrates a 3D diagram of the local support of a bivariate quadratic spline curve, where the provided equidistant grid points are located at *x*_*i* − 1_, …, *x*_*i* + 2_ =  − 1, 0, 1, 2 and *y*_*i* − 1_, …, *y*_*i* + 2_ =  − 1, 0, 1, 2. The bivariate B-spline basis function was connected to all 28 nonzero polynomial surfaces. In addition, it is continuous and has a continuous first-order partial derivative. The following properties were established:

6 $$ {\displaystyle \begin{array}{l}\forall \left(x,y\right)\in D,\kern0.36em \sum {B}_{ij}\left(x,y\right)\equiv 1\\ {}\forall \left(x,y\right)\in D,\kern0.36em \sum {\left(-1\right)}^{i+j}{B}_{ij}\left(x,y\right)\equiv 0\end{array}} $$

The smoothness and local characteristics of the bivariate B-spline make it an ideal candidate as the basis function of the approximation or interpolation kernel.

### Normalized cross-correlation algorithm

Common measures of image similarity include peak signal-to-noise ratio, structural similarity, normalized cross-correlation (NCC), normalized mutual information, and mean square error. Because the calculation of the NCC coefficient is relatively simple, and the reasonable concave-convex characteristic is conducive to solving the optimal parameters, the NCC method was chosen as the similarity measurement algorithm. The NCC similarity metric measures the similarity of the images to be registered by calculating the cross-correlation value between the reference image and the moving image. The NCC of a two-dimensional (2D) image can be expressed as

7 $$ {S}_{\mathrm{NCC}}=\frac{\sum \limits_{i=0}^{m-1}\sum \limits_{j=0}^{n-1}A\left(i,j\right)B\left(i,j\right)}{\sqrt{\sum \limits_{i=0}^{m-1}\sum \limits_{j=0}^{n-1}A{\left(i,j\right)}^2}\sqrt{\sum \limits_{i=0}^{m-1}\sum \limits_{j=0}^{n-1}B{\left(i,j\right)}^2}} $$

where *A*(*i*, *j*) and *B*(*i*, *j*) represent the gray values of the moving image and the reference image in *m* rows and *n* columns, respectively. When the NCC value between the reference and moving images is the largest, the two images are fully registered. Our chosen optimization strategy adopts the gradient descent method to solve the minimum value of the objective function. Therefore, this study selects the reciprocal of Eq. (7).

8 $$ {S}_{NCC}^{\hbox{'}}=\frac{1}{S_{NCC}} $$

### Image registration framework

The 2D arrays *I*_1_(*x*, *y*) and *I*_2_(*x*, *y*) of a known size respectively represent the gray value of the moving and reference images at the point (*x, y*). Subsequently, the registration relationship between the images can be expressed as

9 $$ {I}_2\left(x,y\right)={I}_1\left\{g\left[f\left(x,y\right)\right]\right\} $$

where *f* represents a 2D geometric transformation function, and *g* represents a one-dimensional gray-scale interpolation function.

The geometric transformation function *f* in the non-rigid registration algorithm consists of three parts, including the search space (the non-tensor product B-spline model), similarity measure (the NCC algorithm), and optimization strategy (the gradient descent algorithm). The gray-level interpolation relationship *g* applies a bilinear interpolation algorithm. First, convert the pixel coordinates of the image into parameter grid coordinates, perform the uniform 2-type triangulation on the parameter grid, and calculate the value of the non-tensor product B-spline model transformation. This value is the offset of the image coordinates, each of which is affected by nine control point parameters. Provided that the pixel coordinates of a moving image *I*(*x*, *y*) in row *a* and column *b* are (*x*, *y*) and the parameter coordinates are (*u*, *v*), convert it into a parameter grid with *m* rows and *n* columns, respectively.

10 $$ \left\{\begin{array}{l}u=\left(\frac{x}{a}\cdot m\right)-\left\lfloor \frac{x}{a}\cdot m\right\rfloor \\ {}v=\left(\frac{y}{b}\cdot n\right)-\left\lfloor \frac{x}{b}\cdot n\right\rfloor \end{array}\right. $$

The coordinate offset is as follows:

11 $$ \left\{\begin{array}{l} Tx=\sum \limits_{i=0}^2\sum \limits_{j=0}^2{c}_{i,j}{B}_{i,j}(u)\\ {} Ty=\sum \limits_{i=0}^2\sum \limits_{j=0}^2{c}_{i,j}{B}_{i,j}(v)\end{array}\right. $$

#### Algorithm 1

Pseudo-code implementation of non-tensor product B-spline transformation

**Table:** 

Input:Moving_Image (*x*, *y*), Control_vertex_parameter[2 * m * n]	
Output:Deformation_Image(New_x, New_y)	
1	while x < a and y < b do
2	delta_x = a / m;
3	delta_y = b / n;
4	x_block = x / delta_x;
5	y_block = y / delta_y;
6	w = floor(x_block);
7	s = floor(y_block);
8	u = x_block - w;
9	v = y_block - s;
10	i = 0;
11	j = 0;
12	if (u, v) in the interval of the basis function p(u, v) then
13	While i < 3 do
14	While j < 3 do
15	Tx + = B(u, v) * Control_vertex_parameter[(s + i) * n + w + j];
16	Ty + = B(u, v) * Control_vertex_parameter[(s + i) * n + w + j + m * n];
17	end while
18	end while
19	end if
20	end while
21	New_x = x + Tx;
22	New_y = y + Ty;

In pseudo-code implementations, such as Algorithm 1, the control vertex parameter sequence is first initialized, the parameter sequence and moving image are input into the non-tensor product B-spline model and the pixel coordinates of the moving image are transformed to obtain the deformation image. Subsequently, the NCC algorithm is used to calculate the similarity measure between the deformation and reference images, and then it is judged whether the similarity measure satisfies a certain threshold. If not, the gradient descent algorithm is used to update the control vertex parameters, find the optimal solution using the continuous iteration, and finally export the registration image, as shown in Fig. 6.

**Fig. 6:**
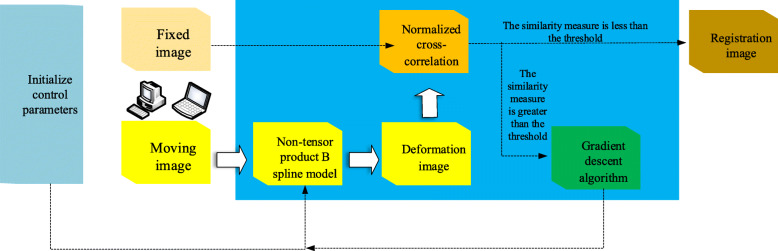
Non-tensor product registration framework

To perform the experiments in this study, an Intel(R) Core (TM) i7-8750H CPU and NVIDIA GeForce GTX 1050 GPU hardware configuration was employed. The graphics processing unit with Compute Unified Device Architecture was used to accelerate the calculation of the non-tensor product B-spline algorithm.

## Results and Discussion

### 33× 33 parameter grid

**Fig. 7:**
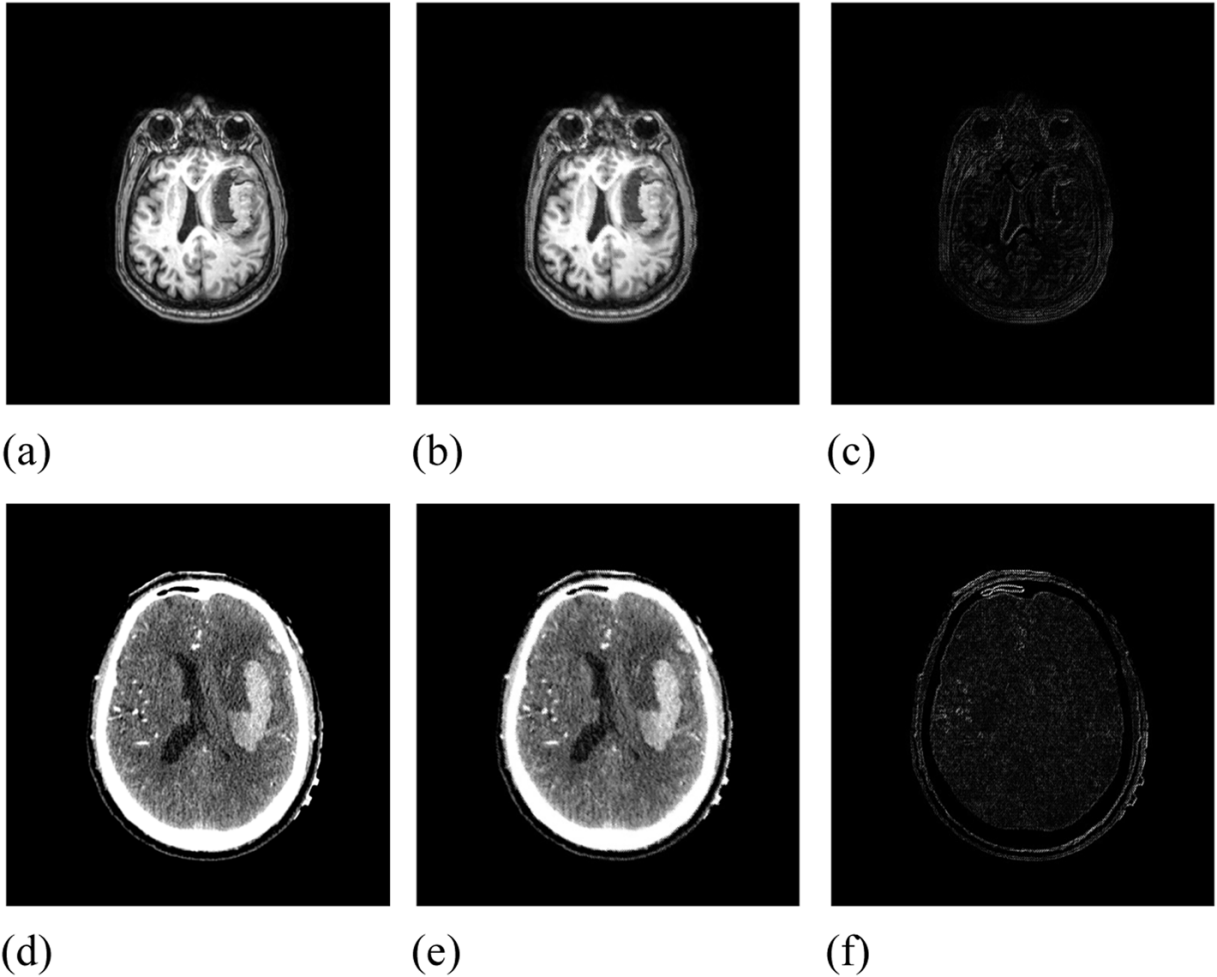
Initial image data. (**a**): MRI reference image; (**b**): MRI moving image; (**c**): Difference between MRI reference image and moving image; (**d**): CT reference image; (**e**): CT moving image; (**f**): Difference between CT reference image and moving image

This group of experiments used MRI and CT images of a patient with a brain hematoma to test the effectiveness of the algorithm in correcting the non-rigid motion of medical images. The size of the images was 496 × 472, and the parameter grid specification was set to 33 × 33, as shown in Fig. 7. The initial similarity measure value of the MRI and CT reference and moving images was 1.01348 and 1.01908, respectively.

#### Tensor product quadratic uniform B-spline

**Fig. 8:**
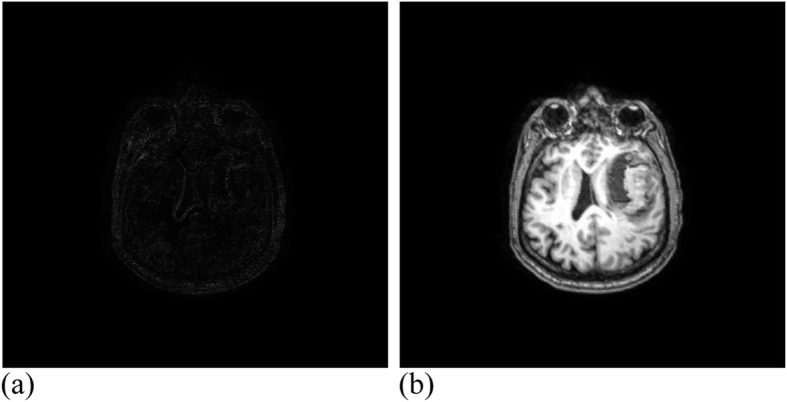
MRI data after registration. (**a**): Difference between MRI reference image and registration image; (**b**): MRI registration image

**Fig. 9:**
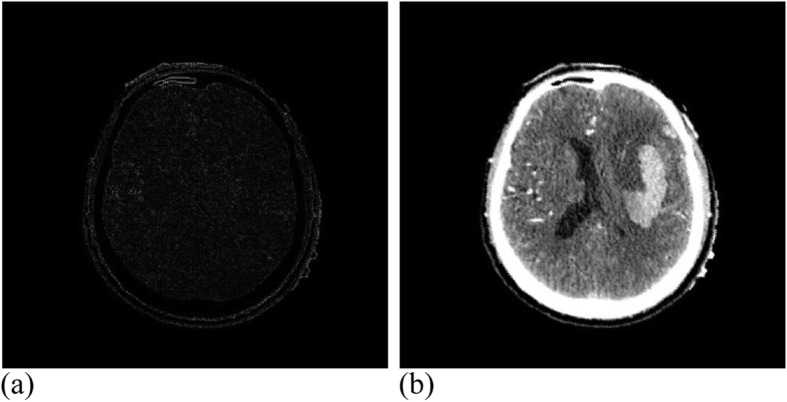
CT data after registration. (**a**): Difference between CT reference image and registration image; (**b**): CT registration image

In this experiment, a two-variable quadratic uniform B-spline tensor product was selected as the deformation function of the registration, in which 3 × 3 control vertices controlled the offset of each pixel. Thirteen valid iterations were performed for the registration experiment of the MRI images. The similarity measure reached 1.00338, where the running time was 5 min. Figure 8 illustrates the MRI experimental results. Eighteen valid iterations were carried out for the registration experiment of the CT images. The final similarity measure, for a running time of 4 min, was 1.00569. Figure 9 demonstrates the CT experimental results.

#### Tensor product cubic uniform B-spline

**Fig. 10:**
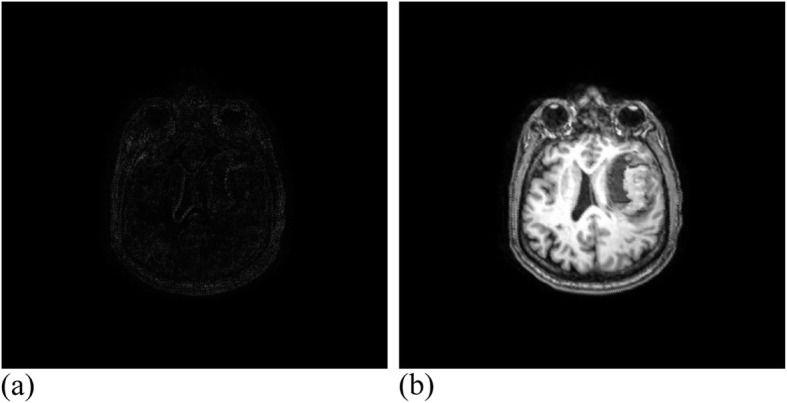
MRI data after registration. (**a**): Difference between MRI reference image and registration image; (**b**): MRI registration image

**Fig. 11:**
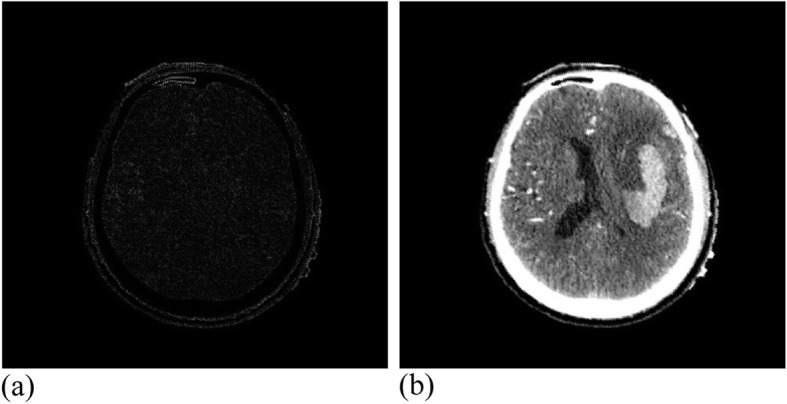
CT data after registration. (**a**): Difference between CT reference image and registration image; (**b**): CT registration image

In this experiment, a two-variable cubic uniform B-spline tensor product was selected as the deformation function of the registration, in which 4 × 4 control vertices controlled each pixel. Sixteen valid iterations were performed for the registration experiment of the MRI images. The similarity measure reached 1.00327, where the running time was 11 min. Figure 10 presents the MRI experimental results. Eighteen valid iterations were carried out for the registration experiment of the CT images. The final similarity measure, for a running time of 10 min, was 1.00559. Figure 11 shows the CT experimental results.

#### Non-tensor product uniform B-spline

In this experiment, a B-spline uniformly divided in the space ${S}_2^1\left({\Delta}_{mn}^{(2)}\right)$ was used, where the node vector was $0,\frac{1}{33},\frac{2}{33},\dots, \frac{31}{33},\frac{32}{33},1$. Here, *m* = 33, *n* = 33; thus, the dimension of ${S}_2^1\left({\Delta}_{33\times 33}^{(2)}\right)$ was 1224, and the set of basis functions to be subtracted was as follows:

12 $$ \left\{\begin{array}{l}\frac{1}{4}{\left(33u+33v-1\right)}^2,0\le u\le \frac{1}{33}\kern0.36em \mathrm{and}\kern0.36em 0\le v\le \frac{1}{33}\kern0.36em \mathrm{and}\kern0.36em u+v-\frac{1}{33}\le 0\\ {}0,\mathrm{others}\end{array}\right\} $$

**Fig. 12:**
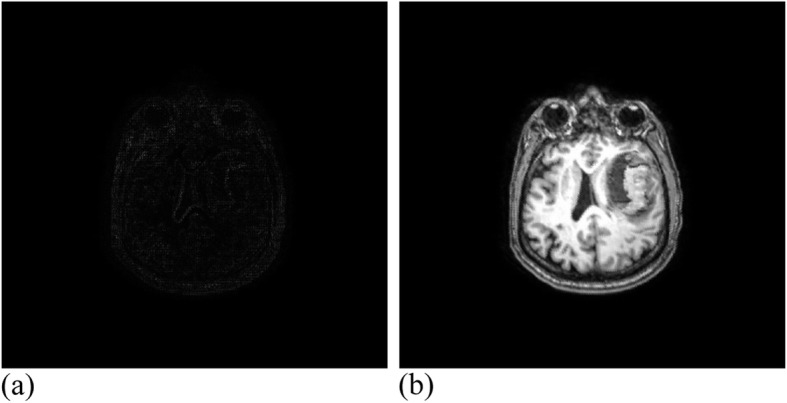
MRI data after registration. (**a**): Difference between MRI reference image and registration image; (**b**): MRI registration image

**Fig. 13:**
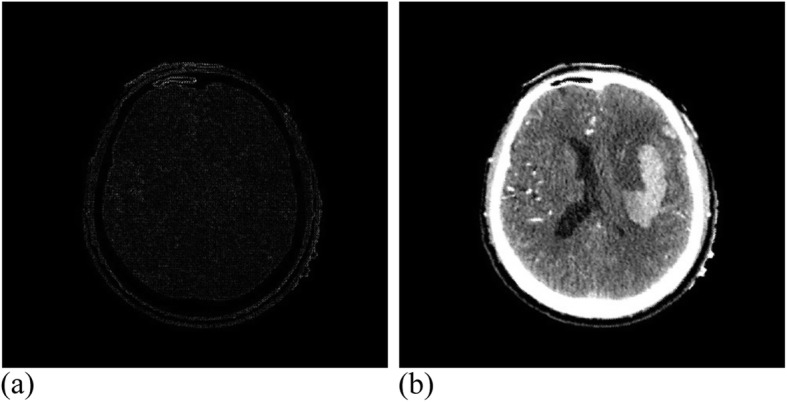
CT data after registration. **(a)**: Difference between CT reference image and registration image; (**b**): CT registration image

Among them, 3 × 3 control vertices controlled the offset of each pixel, where 17 valid iterations were carried out for the registration experiment of MRI images. The similarity measure reached 1.00274, where the running time was 49 min. Figure 12 presents the MRI experimental results. Twenty-eight valid iterations were carried out for the registration experiment of the CT images. The final similarity measure was 1.00457, where the running time was 45 min. Figure 13 illustrates the CT experimental results.

#### Non-tensor product non-uniform B-spline

In this experiment, the non-uniformly divided B-spline in the ${S}_2^1\left({\Delta}_{mn}^{(2)}\right)$ space was used as the deformation function, where the node vector was $0,\frac{1}{33},\frac{3}{33},\frac{4}{33},\dots, \frac{29}{33},\frac{31}{33},\frac{32}{33},1.$ Among them, the 4 × 3 control vertices controlled the pixel offset, and the subtracted set of basis functions was as follows:

13 $$ \left\{\begin{array}{l}\frac{1}{6}{\left(33u+33v-1\right)}^2,0\le u\le \frac{1}{33}\kern0.36em \mathrm{and}\kern0.36em 0\le v\le \frac{1}{33}\kern0.36em \mathrm{and}\kern0.36em u+v-\frac{1}{33}\le 0\\ {}0,\mathrm{others}\;\end{array}\right\} $$

**Fig. 14:**
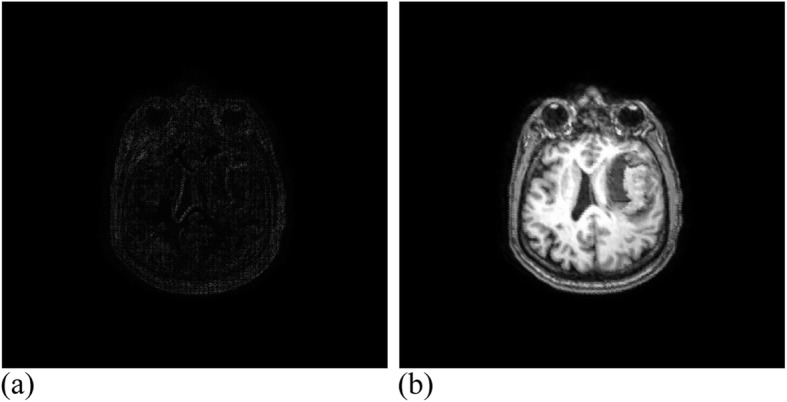
MRI data after registration. **(a)**: Difference between MRI reference image and registration image; (**b**): MRI registration image

**Fig. 15:**
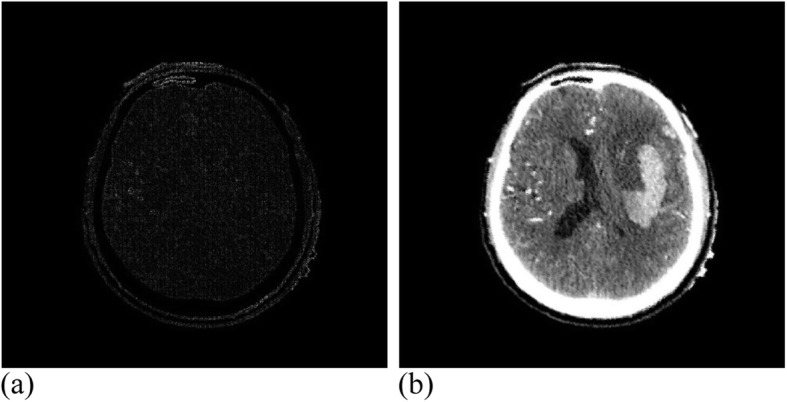
CT data after registration. (**a**): Difference between CT reference image and registration image; (**b**): CT registration image

The MRI registration experiment carried out 19 effective iterations and reached a similarity measure of 1.00537 with a running time of 30 min. Figure 14 presents the MRI experimental results. The CT registration experiment carried out 25 effective iterations, and reached a similarity measure value of 1.00808, where the running time was 28 min. Figure 15 demonstrates the CT experimental results.

**Fig. 16:**
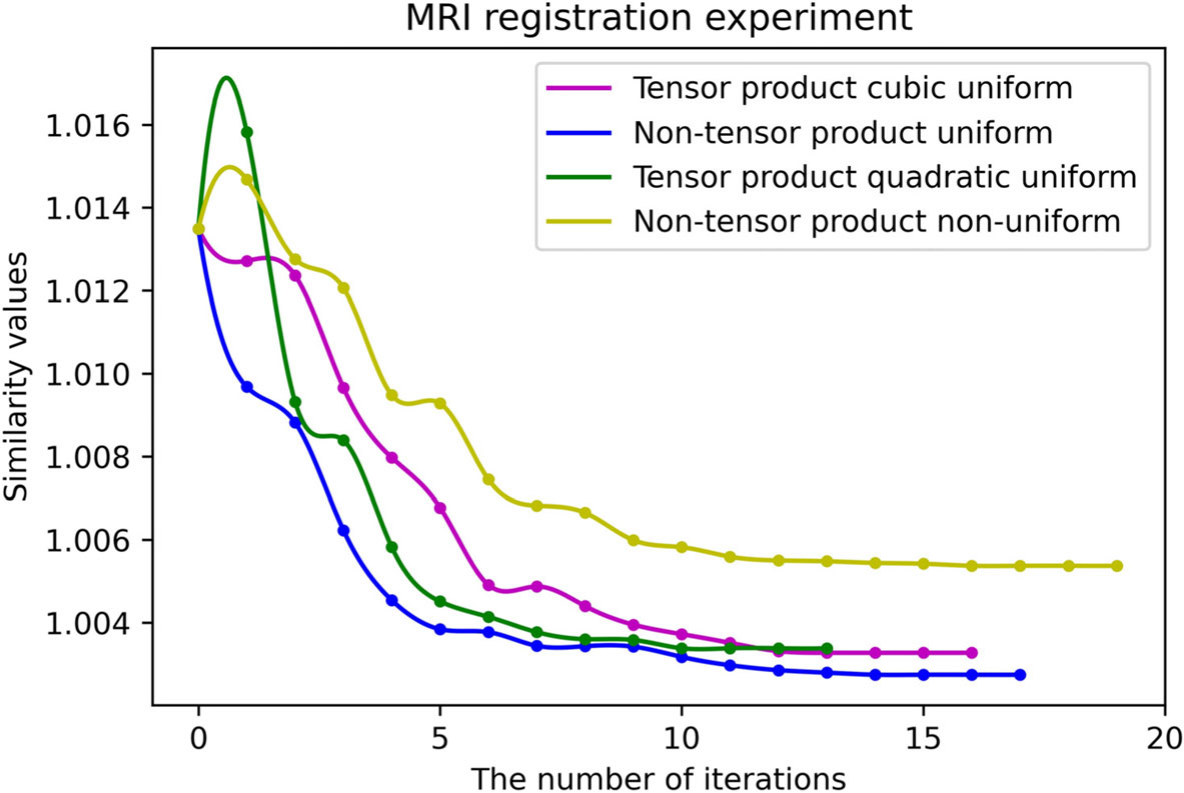
MRI registration experiment

**Fig. 17:**
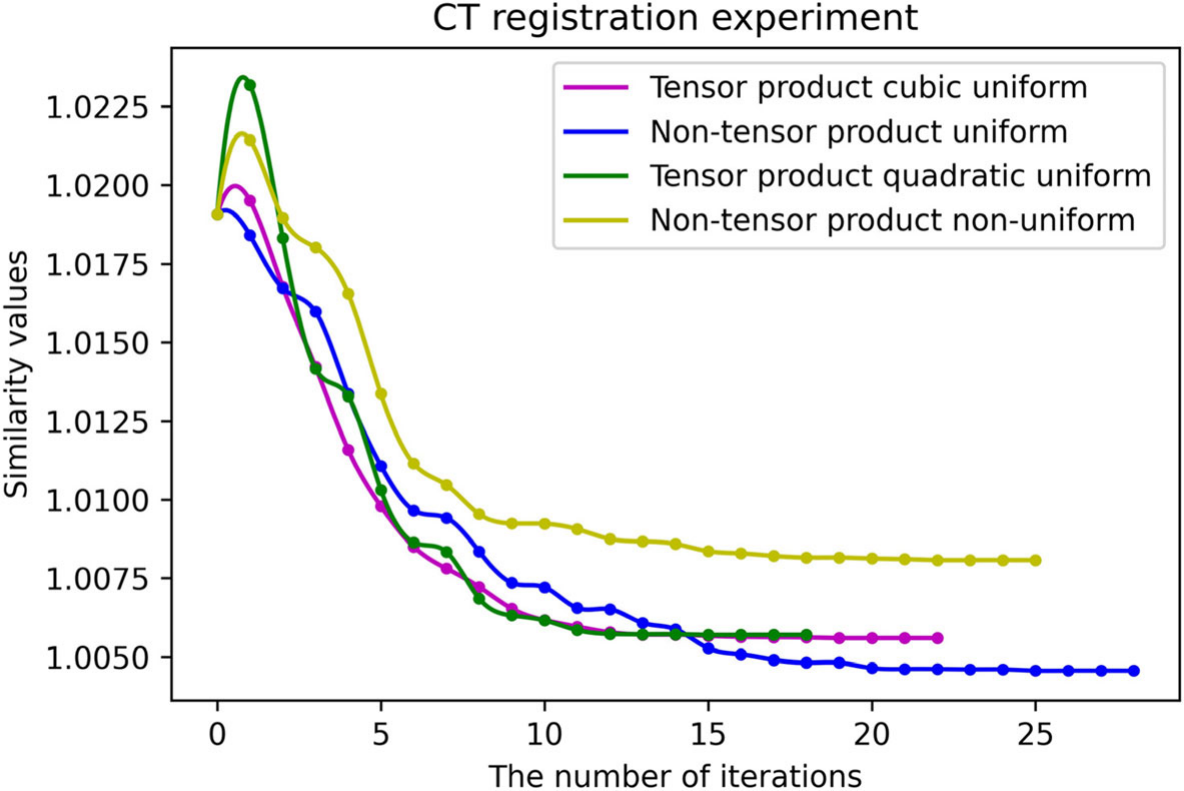
CT registration experiment

Figures 16 and 17 present the fitting curves of the number of iterations and the registration accuracy of different methods in the experimental process, respectively. As shown in Tables 1 and 2, the uniform B-spline method in the non-tensor ${S}_2^1\left({\Delta}_{mn}^{(2)}\right)$ space has the highest registration accuracy.

**Table 1:** MRI registration experiment results

	Similarity measure values	Number of iterations	Running time (min)
Tensor product type quadratic uniform B-spline	1.00338	13	5
Tensor product cubic uniform B-spline	1.00327	16	11
Non-tensor product type quadratic uniform B-spline	1.00874	17	49
Non-tensor product type quadratic non-uniform B-spline	1.00537	19	30

**Table 2:** CT registration experiment results

	Similarity measure values	Number of iterations	Running time (min)
Tensor product type quadratic uniform B-spline	1.00569	18	4
Tensor product cubic uniform B-spline	1.00559	18	10
Non-tensor product type quadratic uniform B-spline	1.00457	28	45
Non-tensor product type quadratic non-uniform B-spline	1.00808	25	28

### 66× 66 parameter grid

**Fig. 18:**
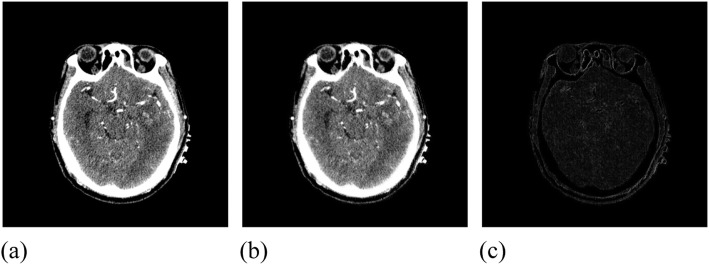
Initial image data. (**a**): CT reference image; (**b**): CT moving image; (**c**): Difference between CT reference image and moving image

The experimental data from this group used CT images of a patient. Its size was 835 × 835, the parameter grid size was 66 × 66, and the node vector was $0,\frac{1}{66},\frac{2}{66},\dots, \frac{64}{66},\frac{65}{66},1$, as shown in Fig. 18. The initial similarity measure of the CT images was 1.01562.

#### Tensor product cubic uniform B-spline

**Fig. 19:**
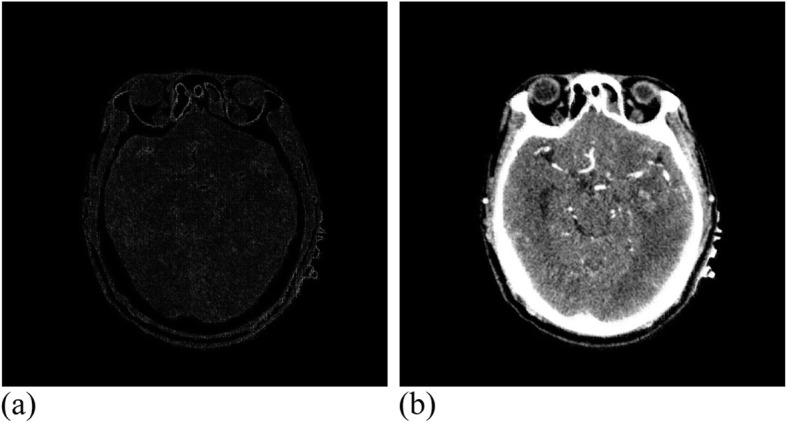
CT data after registration. **(a)**: Difference between CT reference image and registration image; (**b**): CT registration image

This experiment used a tensor product two-variable cubic B-spline as the deformation function. The CT registration experiment carried out 35 effective iterations, and reached a similarity measure value of 1.00671, where the running time was 3 h. Figure 19 presents the experimental results.

#### Non-tensor product uniform B-spline

In this experiment, a B-spline uniformly divided in the ${S}_2^1\left({\Delta}_{mn}^{(2)}\right)$ space was used, and the subtracted set of basis functions was as follows:

14 $$ \left\{\begin{array}{l}\frac{1}{4}{\left(66u+66v-1\right)}^2,0\le u\le \frac{1}{66}\kern0.36em \mathrm{and}\kern0.36em 0\le v\le \frac{1}{66}\kern0.36em \mathrm{and}\kern0.36em u+v-\frac{1}{66}\le 0\\ {}0,\mathrm{others}\end{array}\right\} $$

**Fig. 20:**
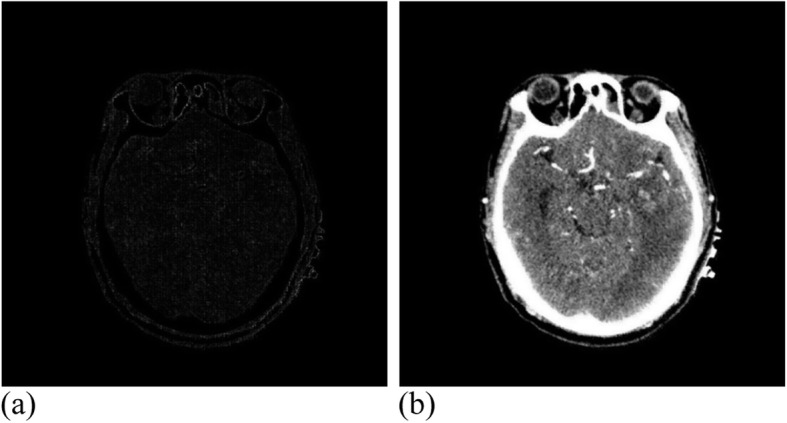
CT data after registration. **(a)**: Difference between CT reference image and registration image; (**b**): CT registration image

The CT registration experiment carried out 36 effective iterations, and reached a similarity measure value of 1.0067, where the running time was 6 h. Figure 20 shows the CT experimental results.

**Fig. 21:**
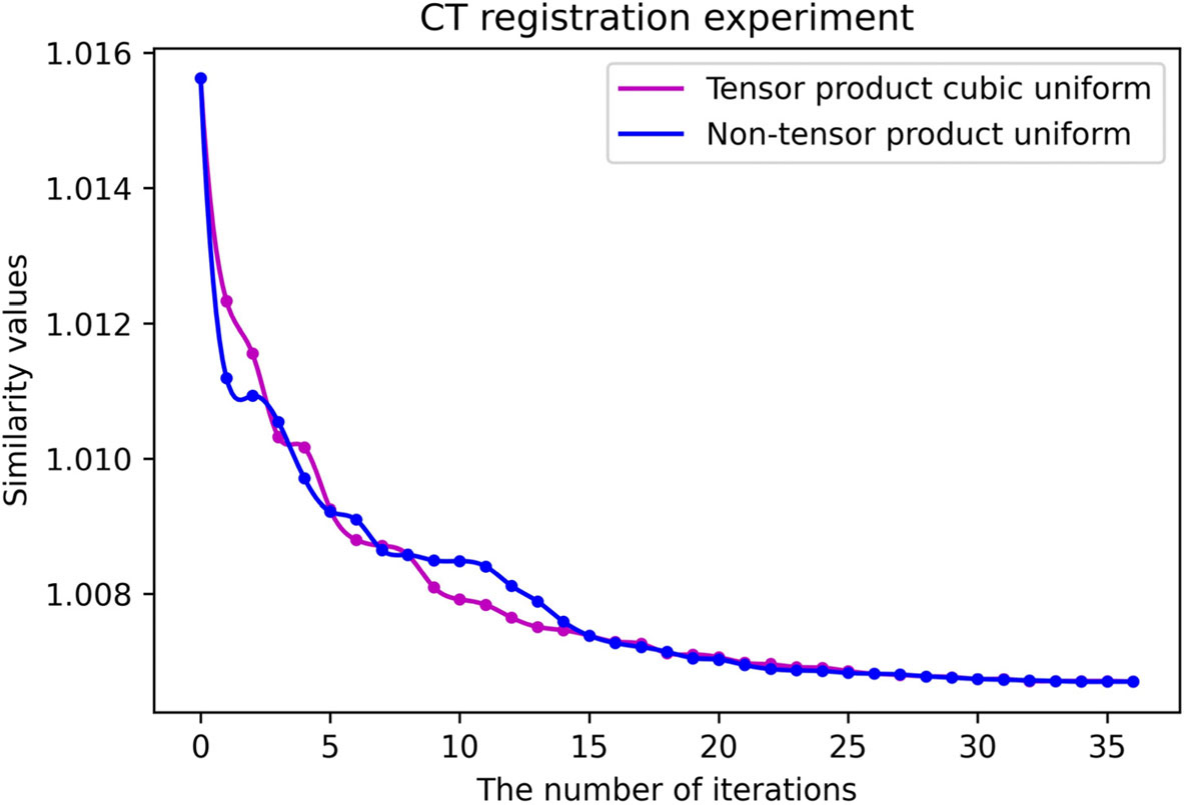
CT registration experiment

Figure 21 illustrates the fitting curves between the number of iterations and the registration accuracy in this set of experiments. Table 3 proves that as the image size and specification of the parameter grid increase, the time cost of the two algorithms increases. Consequently, the experimental results proved that the non-tensor product algorithm obtained a higher accuracy.

**Table 3:** CT registration experiment results

	Similarity measure values	Number of iterations	Running time (h)
Tensor product cubic uniform B-spline	1.00671	35	3
Non-tensor product type quadratic uniform B-spline	1.0067	36	6

## Conclusions

In this study, a non-rigid registration algorithm based on the ${S}_2^1\left({\Delta}_{mn}^{(2)}\right)$ non-tensor-type B-splines was introduced. Applying the proposed algorithm to the search space algorithm could satisfactorily simulate the non-rigid deformation of medical images and describe the dynamic motion of medical images. Indeed, the ${S}_2^1\left({\Delta}_{mn}^{(2)}\right)$ non-tensor product B-spline algorithm is a function defined in four directions. Compared with the tensor product spline function, it can describe the deformation of the image in more directions. Simultaneously, the ${S}_2^1\left({\Delta}_{mn}^{(2)}\right)$ non-tensor product B-spline algorithm is highly flexible in the processing of boundary triangles. By comparing the performance of different methods, the non-tensor product uniform B-spline algorithm yielded the highest accuracy. The errors in this study might have been caused by the slicing technology or tissue effects, which will be attempted to be reduced in the future studies. Although the accuracy could be improved, the required time was increased owing to the increased complexity of the algorithm. This problem can be addressed by changing the optimization algorithm of the search space and increasing the computing power of the device. However, further study is required to investigate the model’s parametric mesh subdivision and convergence.

## Data Availability

The datasets used and/or analyzed during the current study are not publicly available due to personal privacy, but are available from the corresponding author upon reasonable request.

## Abbreviations

CT: Computed tomography
MRI: Magnetic resonance imaging
3D: Three-dimensional
PET: Positron emission computed tomography
US: Ultrasound
2D: Two-dimensional
NCC: Normalized cross-correlation
